# Outcome Improvement with Last-Generation Devices in Mitral Transcatheter Edge-to-Edge Repair: Insights from the Real-World MitraClip Florence Registry

**DOI:** 10.3390/jcm14041075

**Published:** 2025-02-08

**Authors:** Mattia Alexis Amico, Sabato Tedesco, Chiara Piazzai, Guido Grossi, Gherardo Busi, Giorgia Panichella, Angela Migliorini, Francesco Meucci, Renato Valenti, Carlo Di Mario, Nazario Carrabba

**Affiliations:** Cardio-Thoraco-Vascular Department, Careggi Hospital, 50134 Florence, Italy

**Keywords:** mitral regurgitation, transcatheter edge-to-edge repair, MitraClip, valvular heart disease

## Abstract

**Background/Objectives:** Over the past two decades, MitraClip™ therapy has proven to be an effective and safe treatment for severe mitral regurgitation (MR), with more than 200,000 patients treated globally through continuous advancements in device design and implantation techniques. This retrospective, observational, single-center study aimed to assess the safety and efficacy of the latest generation of MitraClip compared to earlier models in the Real-World MitraClip Florence Registry. The primary efficacy endpoint was a comparison in terms of the rate of successful procedures, the time to device deployment and the duration of the hospital stay. The secondary safety endpoint regarded long-term all-cause mortality and hospitalization for heart failure. **Methods**: Patients treated at our center from January 2016 to June 2022 were included. They were divided into two groups: those receiving early-generation devices (G1–G3) and those treated with the last-generation device (G4). All patients underwent a comprehensive preoperative echocardiographic assessment, with a re-evaluation before hospital discharge and after 12 months. A long-term follow-up focusing on all-cause mortality and hospitalization for heart failure was conducted. **Results**: Of 131 patients, 81 received the last-generation device. The mean age was 79.4 years. Both groups exhibited a high burden of comorbidities (overall mean *n* = 2.85). Procedural success was high (97%) across groups, with a significantly better MR reduction (Grade ≤ 1) in the G4 group (47% vs. 70%, *p* = 0.009). The time to device deployment was significantly shorter with the G4 system (72 vs. 135 min, *p* < 0.001), and there was a trend towards shorter hospital stays (6.1 vs. 7.9 days, *p* = 0.08). Kaplan–Meier analysis demonstrated better 5-year survival rates for the last-generation device group (*p* = 0.019), with no significant difference in rehospitalization rates (*p* = 0.186). **Conclusions**: The MitraClip G4 system in the real world for the treatment of severe MR is safe and effective, achieving immediate and durable procedural success, accompanied by an improved NYHA functional class. Moreover, a better long-term survival rate was observed, along with a comparable high rate of recurrent HF hospitalization, reflecting a high comorbidity burden in this frail population.

## 1. Introduction

Over the past 20 years, MitraClip™ therapy (Abbott Cardiovasular, Green Oaks, IL, USA) has demonstrated efficacy and safety in numerous clinical studies, with more than 200,000 patients treated worldwide [1,2]. The safety and effectiveness of early-generation (G1, G2) mitral transcatheter edge-to-edge repair (M-TEER) devices were established in historical trials and registries [3,4,5,6]. The third-generation M-TEER device introduced a more precise and predictable delivery system (NTR), as well as longer clip arms (XTR), improving ease of use, enabling the treatment of a broader range of anatomies, and showing better outcomes in the real-world EXPAND study [7] compared to the first-generation devices. The current fourth-generation M-TEER device, the MitraClip G4 system, showed an improved clip deployment system capable of continuously monitoring left atrial pressure for real-time hemodynamic monitoring, an independent and controlled gripper actuation, and two additional wider clip sizes, with 9 mm (NTW) and 12 mm (XTW) arm lengths. The safety and effectiveness of the MitraClip G4 was demonstrated by the EXPAND G4 study both at 30 days and the 1-year follow-up [8]. The aim of this study is to evaluate the efficacy and the safety of the last generation of MitraClip in comparison to previous versions in the Real-World MitraClip Florence Registry.

## 2. Materials and Methods

### 2.1. Design and Study Population

All patients with severe mitral regurgitation who underwent TEER at our tertiary center between January 2016 and June 2022 were retrospectively enrolled. All TEER procedures were discussed and approved by our local Heart Team. Patients were divided into two groups based on the device used: the first group included patients treated with first-, second- and third-generation devices (early-generation device group [EarlyGen]: MitraClip G1, G2, G3—NT, NRT, XTR), and the second group comprised patients treated with fourth-generation devices (last-generation device group [LastGen]: MitraClip G4 system—NTR/NTW, XTR/XTW). Data on demographics, cardiovascular risk factors, and comorbidities were extracted from clinical records. Information about the urgency of the procedure (urgent or non-urgent), the type of device implanted, time to device deployment, and intraoperative complications was obtained from the operative reports. Urgent treatment was defined as the need for percutaneous intervention during index hospitalization, while in other cases, the procedure was scheduled for a subsequent hospitalization. Time to device deployment was defined as the time from the initiation of the procedure until the device was successfully deployed. The presence of complications during hospitalization was assessed according to the Mitral Valve Academic Research Consortium (MVARC) [9]. Follow-up information on the occurrence of death or hospitalization for heart failure was obtained through clinical visits or phone interviews. The ethics committee of Careggi University Hospital approved the study protocol, which conforms to the Declaration of Helsinki [10], and all patients provided written informed consent.

### 2.2. Echocardiographic Examinations

All patients underwent a comprehensive preoperative echocardiographic assessment, followed by a thorough re-evaluation before hospital discharge. A follow-up echocardiographic evaluation was performed 12 months after the procedure. All echocardiographic examinations were conducted in accordance with the American Society of Echocardiography guidelines, using a validated multi-integrative method [11,12]. Both qualitative (CFM: color flow mapping) and quantitative (PISA: proximal isovelocity surface area) measurements were used to grade MR severity from Grades 0 to 4 (Grade 0: no trace; Grade 1: mild; Grade 2: moderate; Grade 3: moderate-to-severe; and Grade 4: severe). Systolic pulmonary arterial pressure (PAPs) was calculated by summing the trans-tricuspid regurgitation gradient and the estimated central venous pressure. Procedural success was defined as the uncomplicated placement of ≥1 clip, resulting in a peri-procedural estimated MR reduction to ≤Grade 2 in severity, in accordance with the MVARC criteria [9].

### 2.3. Clinical Follow-Up

All patients underwent clinical follow-up to assess symptoms, rehospitalization and vital status. Data were extracted from clinical registries where accessible, and through follow-up planned visits. For patients with missing details, information was obtained via phone calls to the patients themselves or to a caregiver.

### 2.4. Endpoints

The primary efficacy endpoint of the study was a comparison between early-generation and last-generation devices in terms of the rate of successful procedures. An additional efficacy endpoint was the time taken for device placement (time to device deployment) and the duration of the hospital stay. The secondary safety endpoint analyzed the difference in long-term all-cause mortality and hospitalization for heart failure (HF) between the two groups. HF-related hospitalizations were defined as total worsening heart failure events, including both first and recurrent unplanned hospitalizations.

### 2.5. Statistical Analysis

Continuous variables were expressed as mean ± standard deviation, and categorical variables as frequency and percentage. Distribution normality was assessed using the Shapiro–Wilk test. Differences between patients in the two groups were compared using Student’s *t*-test or a Wilcoxon rank-sum test for continuous variables, and a Chi-square or Fisher’s exact test for categorical variables, as appropriate. The cumulative incidence of individual outcomes was estimated using the Kaplan–Meier method and compared using a log-rank test. Statistical analysis was performed using SPSS version 29.0. A two-sided *p*-value of less than 0.05 was considered statistically significant.

## 3. Results

### 3.1. Baseline and Medical History

A total of 131 patients were retrospectively enrolled. Among them, 50 were treated with early-generation devices, and 81 with last-generation devices. The last-generation devices were implanted from July 2019 onwards. Table 1 shows the clinical and demographic characteristics of the patient groups. No significant differences were observed between the two groups regarding age and traditional risk factors; however, diabetes mellitus and dyslipidemia were more prevalent in the Early-Gen device group. Atrial fibrillation was more prevalent in the Last-Gen device group. Both groups of patients exhibited a high burden of comorbidities, with no significant differences between the two groups.

### 3.2. Preoperative Echocardiographic Examination

Preoperative echocardiographic data (Table 2) confirmed the presence of severe mitral regurgitation in all patients, with no differences in terms of EROA of MR between the two groups (0.36 ± 0.07 cm^2^ vs. 0.35 ± 0.07 cm^2^, *p* = 0.39). In both groups, functional etiology of mitral regurgitation was the most frequent (72.5% vs. 58.8%), followed by primary forms (27.5% vs. 41.2%), with no statistical differences between the two groups (*p* = 0.108). All patients exhibited ventricular dysfunction (36.4% vs. 39.9%, *p* = 0.196), with a trend toward greater left ventricular dilation in the Early-Gen device group (end-diastolic diameter [EDD] = 59.9 mm vs. 57.2 mm, *p* = 0.09). In our population, 67 patients (51.1%) had a COAPT-like profile (secondary severe MR with EF 20–50% and EDD ≤ 70 mm)—29 (56.7%) in the EarlyGen group and 38 (46.9%) in the LastGen group—with no significant differences (*p* = 0.779). Analysis of the right heart sections showed normal right ventricular function in both groups (mean TAPSE of 18.6 mm vs. 17.8 mm) with comparable tricuspid regurgitation severity (graded 2.1 ± 0.8 vs. 2.2 ± 0.9). The evaluation of mean pulmonary artery systolic pressure showed similar values in the two groups (37.3 mmHg vs. 35.1 mmHg, *p* = 0.355).

### 3.3. Procedural Data

**Figure 1:**
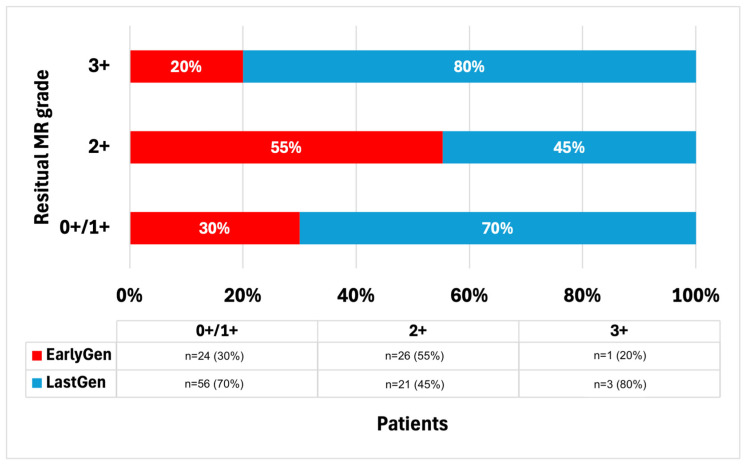
Residual post-procedural MR degrees.

Overall, 49 patients required the implantation of more than one device, with no differences between the early and last generations (*n* = 18 [36%] vs. *n* = 31 [38.3%], *p* = 0.628). In the last-generation group, the majority (*n* = 74 [91.4%]) received at least an XTR/XTW device, while seven patients (8.6%) received only an NTR/NTW device. A summary of the residual post-procedural MR degrees is presented in Figure 1. Overall, the procedural success rate was 97% (127 out of 131 procedures). Specifically, a residual trivial MR (grade 0+/1+) was observed in 61% (*n* = 80) of the patients, significantly higher in the Last-Gen device group (24 [47%] vs. 56 [70%], *p* = 0.009). No differences were observed in terms of residual MR grade ≤2+ between the two groups (50 vs. 77 patients, *p* = 0.562), or in the residual moderate-to-severe MR (grade 3+/4+; *n* = 1 vs. 3, *p* = 0.423). The post-procedure MV gradients were significantly lower in the Last-Gen device group (3.92 ± 2.19 mmHg vs. 2.78 ± 1.29 mmHg, *p* < 0.001).

**Figure 2:**
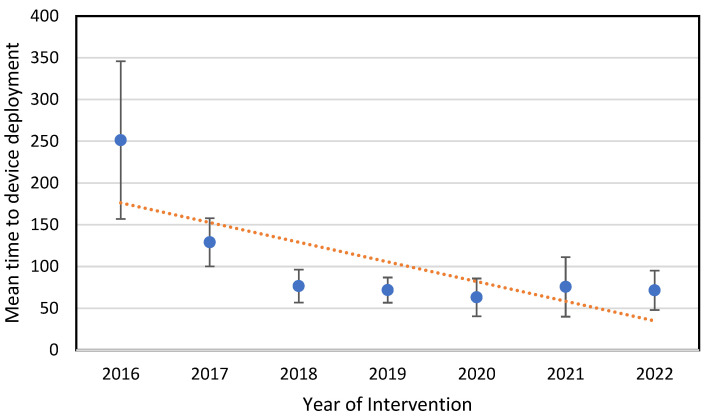
Mean time to device deployment comparison throughout the years.

A statistically significant reduction in the time to device deployment was observed in the Last-Gen device group (135.3 ± 6.1 min vs. 72 ± 5.2 min; *p* < 0.001). Moreover, the time to device deployment tended to shorten year by year (see Figure 2).

### 3.4. In-Hospital Data

In our registry, nearly a quarter of the patients underwent urgent procedures (28, 22.9%) with a significantly higher percentage in the Last-Gen group (2 vs. 26; *p* = 0.001). The length of hospital stay showed a trend toward reduction in the Last-Gen device group, although the difference was not statistically significant (7.9 ± 6.4 vs. 6.1 ± 5.2 days, *p* = 0.08). In-hospital complications according to the MVARC criteria occurred in 34 patients (24.5% of the total), 15 in the Early-Gen group (30%) and 19 in the Last-Gen group (21.3%), with no statistically significant difference (*p* = 0.786). Bleeding requiring blood transfusion, initiated when the hemoglobin concentration dropped below 7 g/dL or based on the physician’s clinical judgment, was the most common complication observed in both the Early-Gen and Last-Gen device groups (6 [11.8%] vs. 12 [15%]). All bleeding events occurred acutely during the hospital stay and were related to access-site complications, requiring surgical revision in only two patients from the Early-Gen group. The second most common complication was arrhythmias (1 [2%] vs. 4 [4.9%]), which included either supraventricular or non-sustained ventricular types treated with medical therapy. There were no intra-procedural deaths, but five in-hospital deaths occurred during urgent procedures, all in patients with cardiogenic shock: two in the Early-Gen group vs. three in the Last-Gen device group (3.9% vs. 3.7%, respectively). Drug therapy at discharge is summarized in Table 3. Notably, the Last-Gen device group had a higher rate of ARNI prescriptions (*p* = 0.035) and a higher rate of DOAC prescriptions, likely due to their greater proportion of patients with atrial fibrillation (*p* = 0.010).

### 3.5. Clinical Follow-Up

**Figure 3:**
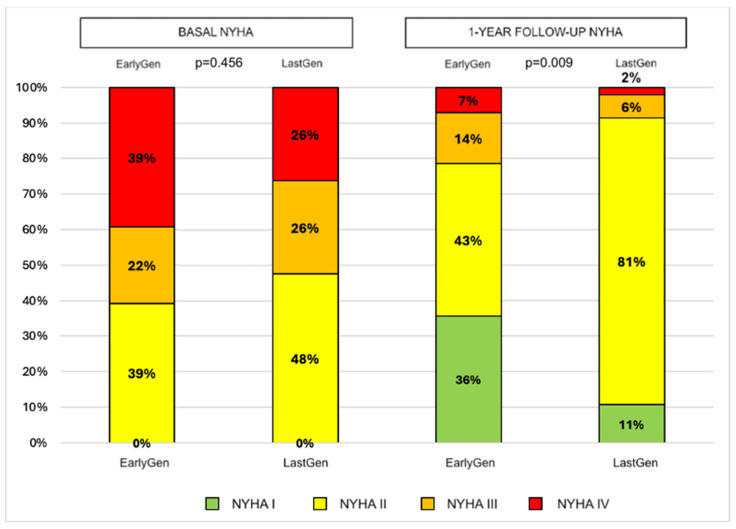
Changes in NYHA functional class according to device generation from baseline to 1-year follow-up.

Through 1 year of follow-up, the death rates were similar between the two groups (10 vs. 18, *p* = 0.836), as were the 1-year hospitalization rates (16 vs. 15, *p* = 0.190). In the surviving patients, no significant changes in the degree of residual MR were observed in either device group (mean residual MR degree: 1.1 ± 0.5 vs. 1.03 ± 0.7, *p* = 0.676), with consistent results compared to the post-operative evaluation. A better 1-year NYHA functional class was observed in the Last-Gen device group (*p* = 0.009; see Figure 3).

**Figure 4:**
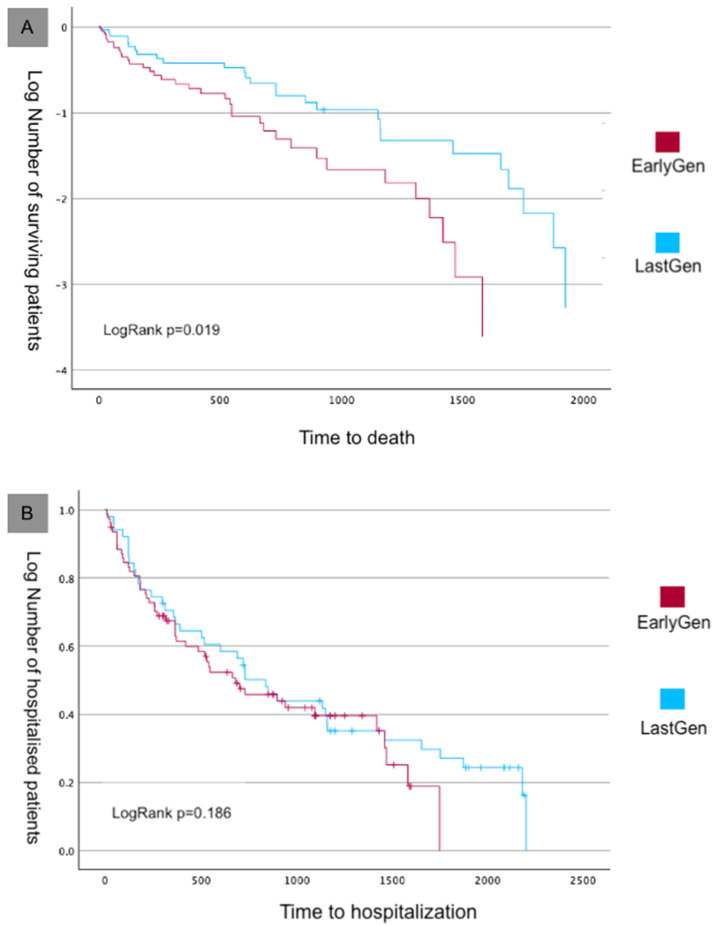
Kaplan–Meier survival analysis of the primary endpoint. (**A**) Survival curves for all-cause mortality. (**B**) Rehospitalization curves.

During the long-term follow-up (5.1 ± 1.2 years), the rate of mortality continued to increase in both groups. Overall, at the end of follow-up, 67 deaths from all causes occurred (51.1% of the entire population): 30 deaths in the Early-Gen device group and 37 deaths in the Last-Gen device group (58.9% vs. 45.7% of the initial group size, respectively). According to the Kaplan–Meier analysis, the survival curves were better in the Last-Gen device group (Figure 4A; Log Rank *p* = 0.019). The total number of worsening heart failure events was 95, with 50 in the Early-Gen group and 45 in the Last-Gen device group (*p* = 0.21), with no differences in the Kaplan–Meier curves (Figure 4B; Log Rank *p* = 0.186).

## 4. Discussion

Our registry reports on the procedural success and long-term outcomes of patients treated with Early-Gen compared to those treated with Last-Gen TEER devices at our tertiary center. After the TEER procedure, a greater reduction in MR was observed with the Last-Gen device, with more than half of the patients achieving no MR or trace MR (grade 0+/1+; *n* = 56, 70%) and 97% achieving procedural success. Furthermore, a significant reduction in time to device deployment was observed with the Last-Gen device, along with a trend toward a reduced length of hospital stay. The 1-year follow-up showed persistent reduction in MR, leading to an improved NYHA functional class. The 5-year all-cause mortality rate was lower in the Last-Gen device group, but similar rates of hospitalization for heart failure (HF) were observed between the two groups. Our data support the effectiveness and long-term safety of the Last-Gen TEER device in the treatment of primary and secondary mitral regurgitation in the real world.

MR reduction to ≤2+ has been the standard for TEER with early-generation devices. Historical studies reported high rates of MR reduction to ≤2+ in the EVEREST II randomized controlled trial (81.6%) [4], the EVEREST II High-Risk Registry and REALISM Continued Access Study High-Risk Arm (83.6%) [2], and the COAPT trial (94.8%) [6]. However, MR reduction to ≤1+ was noticeably lower in these studies, ranging from 36.9% to 69.1% [2,6]. In our registry, the 70% rate of MR reduction to ≤1+ using the Last-Gen device for TEER is remarkable, although it is less than the 92.6% reported in the EXPAND G4 study. Additionally, the post-procedural MV gradients were lower in the Last-Gen device group compared to those observed in the Early-Gen device group. Thus, our data, in agreement with the EXPAND G4 study, demonstrate a continuous improvement in TEER therapy during hospital stay.

The data of our registry showed that the 1-year mortality and total HF hospitalization (HF-H) rates was 21% and 23%, respectively. These findings are in agreement with historical studies, which reported 1-year all-cause mortality rates of 22.8% in the EVEREST II High-Risk Registry and REALISM study [13], 15.3% in the TVCT registry, 19.8% in TRAMI [5], 24.3% in MITRAFR [14], and 19.1% in COAPT [6]. With the third-generation M-TEER device in EXPAND, the 1-year all-cause mortality and HF hospitalization rates were 14.9% and 18.9%, respectively [7]. With the fourth-generation M-TEER (EXPAND G4 trial), the data on mortality and hospitalization were even lower than those reported in previous studies, at 12.3% and 16.9%, respectively [15]. Thus, it is conceivable that continuous improvement in technical and medical skills for these procedures may lead to further improvements in outcomes. Moreover, the latest RESHAPE HF study [16] suggests that the beneficial effect of MitraClip extends to patients with moderate, as well as severe, MR. Importantly, despite the encouraging results from some registries [17], the beneficial effect of treating MR in cardiogenic shock patients remains to be demonstrated, as all five in-hospital deaths in our registry occurred within this specific setting.

The Kaplan–Meier 5-year mortality rate in our registry was lower in the Last-Gen device group, though no difference was observed in the 5-year HF hospitalization rate between the two groups. In the COAPT trial, which involved patients with heart failure and severe secondary mitral regurgitation who remained symptomatic despite maximal medical therapy and other indicated treatments, the TEER procedure resulted in lower rates of hospitalization for heart failure and reduced all-cause mortality over 5 years [18]. Despite the favorable risk–benefit profile of mitral TEER, adverse outcomes continued to occur in both groups, with 73.6% of patients in the device group and 91.5% in the control group either dying or being hospitalized for heart failure within 5 years. Our findings, involving either primary and secondary MR, confirm the high rates of mortality and hospitalization for heart failure observed in long-term follow-up, but with a more favorable survival rate for the Last-Gen device group.

Several factors may contribute to these enhanced outcomes. First, the G4 devices are technically easier to use, featuring separate grasping mechanisms that facilitate the procedure and larger clip sizes that simplify deployment and improve device performance Additionally, as all the procedures were performed by a pool of expert interventional cardiologists with a progressive learning curve, enhanced operator experience could play a role. Moreover, advances in medical therapy, such as the use of ARNI, particularly among patients in the Last-Gen device group, could further enhance these results. The effectiveness of ARNI in heart failure has been well demonstrated in pivotal studies [19], and more evidence suggests that ARNI may also reduce functional mitral regurgitation in patients with heart failure with reduced ejection fraction by lowering ventricular stress and promoting cardiac remodeling [20,21]. In our population, the extent to which the increased use of sacubitril–valsartan during follow-up contributed to improved outcomes in the Last-Gen device group remains uncertain, although some beneficial effect is reasonable. In addition, SGLT2i was only available during the final phase of enrollment, which explains its low use in our population and hindered an evaluation of their impact on outcomes [22].

Finally, it is important to recognize that cardiovascular and non-cardiovascular events requiring hospitalization continued to occur over time, even after successful TEER. This finding reflects the advanced age and multiple coexisting conditions in both groups within our study population. Given the relatively small sample size and the absence of a control group, assessing the specific impact of differing factors on outcomes remains challenging. The COAPT trial clearly demonstrated the beneficial effect of MitraClip therapy in both patients with and without AF and diabetes. Additionally, in our previous study [12], diabetes was not found to significantly impact outcomes in patients undergoing MitraClip implantation. These findings highlight the need for better patient selection and additional therapies to address the various comorbidities in this high-risk population.

## 5. Limitations

Several limitations affect the present registry, with the small sample size being the first and most significant. However, unlike RCT studies, our less restrictive criteria for TEER allowed us to evaluate a more diverse, real-world population encountered in clinical practice. Second, the lack of an independent clinical-events committee and an echocardiographic core laboratory may increase the variability in our data assessment. Nevertheless, all echocardiograms were performed by expert cardiologists with extensive expertise to minimize potential bias. Third, enhanced operator experience during this time could play a significant role.

Notwithstanding these limitations, hospitalizations for heart failure were adjudicated only when strict criteria were met, and the reduction in all-cause mortality over 5 years of follow-up provides reassurance regarding the use of the Last-Gen device for TEER in clinical practice. These findings reflect treatment with the Last-Gen device at our tertiary center, so caution is necessary in extending our results to other populations living in different countries and with different characteristics of mitral regurgitation.

## 6. Conclusions

Our data showed that the use of the fourth-generation MitraClip device in the real world for the treatment of severe MR is safe and effective, achieving immediate and durable procedural success, accompanied by an improved NYHA functional class. Moreover, a better long-term survival rate was observed using the fourth-generation MitraClip, along with a similar high rate of recurrent HF hospitalization, reflecting a high comorbidity burden in this frail population.

## Figures and Tables

**Table 1:** Demographics and cardiovascular risk factors of the population. Continuous variables are presented as the mean ± standard deviation, while categorical variables are presented as counts and percentages. Significant *p*-values are highlighted in bold. BMI: body mass index.

	All Patients (*n* = 131)	EarlyGen (*n* = 51)	LastGen (*n* = 80)	*p* Value
Age, years	79.4 ± 9.1	80.1 ± 7.3	79.4 ± 9.0	0.646
Female, *n* (%)	82, 62.6%	28, 54.9%	54, 67.5%	0.945
Number of comorbidities, *n*	2.85 ± 1.53	3.02 ± 1.24	2.85 ± 1.53	0.509
Smokers, *n* (%)	42, 32.1%	13, 25.5%	29, 36.3%	0.198
Dyslipidemia, *n* (%)	70, 53.4%	34, 66.7%	36, 45.0%	**0.032**
Diabetes, *n* (%)	31, 23.7%	19, 37.3%	12, 15.0%	**0.003**
Hypertension, *n* (%)	92, 70.2%	36, 70.6%	56, 70.0%	0.198
Obesity (BMI > 30)	12, 9.2%	7, 13.7%	5, 1.0%	0.148
Atrial fibrillation, *n* (%)	76, 58.0%	24, 47.1%	52, 65.0%	**0.042**
Chronic renal failure, *n* (%)	65, 26.7%	26, 51.0%	39, 48.8%	0.803
Coronary artery disease, *n* (%)	76, 58.0%	29, 56.9%	47, 58.8%	0.831
Anemia, *n* (%)	51, 39.0%	25, 49.0%	26, 32.5%	0.059

**Table 2:** Preoperative echocardiographic findings. Continuous variables are presented as the mean ± standard deviation, while categorical variables are presented as counts and percentages. EDD: end-diastolic diameter; EROA: effective regurgitant orifice area; LV EF: left ventricle ejection fraction; MR: mitral regurgitation; PAPs: pulmonary artery pressure; TAPSE: tricuspid annular plane systolic excursion; TR: tricuspid regurgitation.

	All Patients (*n* = 131)	EarlyGen (*n* = 51)	LastGen (*n* = 80)	*p* Value
Primary MR, *n* (%)	47, 35.9%	14, 27.5%	33, 41.2%	0.108
Secondary MR, *n* (%)	84, 64.1%	37, 72.5%	47, 58.8%	0.108
Mean EROA, cm	35.9 ± 0.07	0.36 ± 0.07	0.35 ± 0.07	0.390
EDD, mm	58.1 ± 8.8	59.8 ± 8.1	57.1 ± 9.1	0.090
LV EF, %	38.5 ± 14.8	36.4 ± 13.3	39.9 ± 15.7	0.196
TAPSE, mm	18.1 ± 3.7	18.6 ± 3.9	17.8 ± 3.6	0.225
TR degree, *n* (%)	2.1 ± 0.8	2.1 ± 0.8	2.2 ± 0.9	0.802
PAPs, mmHg	35.9 ± 12.1	37.3 ± 12.0	35.1 ± 12.1	0.355

**Table 3:** Drug therapy at discharge.

	All Patients (*n* = 131)	EarlyGen (*n* = 51)	LastGen (*n* = 80)	*p*-Value
ASA *n*, %	45, 34.3%	19, 37.2%	26, 32.5%	0.642
DAPT *n*, %	66, 50.4%	21, 41.2%	45, 56.3%	0.069
VKA *n*, %	28, 21.4%	14, 27.5%	14, 17.5%	0.197
DOAC *n*, %	42, 32.1%	10, 19.6%	32, 40.0%	**0.010**
ACEi/ARBs *n*, %	52, 40.0%	24, 47.1%	28, 35.0%	0.199
ARNI *n*, %	21, 16.0%	4, 7.8%	17, 21.1%	**0.035**
CCB *n*, %	5, 3.8%	2, 3.9%	3, 3.8%	0.959
BBs *n*, %	98, 74.8%	35, 68.6%	63, 78.8%	0.093
SGLT2i *n*, %	3, 2.3%	0, 0%	3, 3.8%	0.156
Loop diuretics *n*, %	117, 89.3%	48, 94.1%	69, 86.3%	0.160
TZDs *n*, %	4, 3.1%	2, 3.9%	2, 2.5%	0.624
Statins *n*, %	68, 51.9%	32, 62.7%	36, 72.0%	0.058
PPI *n*, %	55, 42.0%	19, 37.3%	36, 45.0%	**<0.001**

Significant *p*-values are highlighted in bold. ASA: Acetylsalicylic Acid; ACEi: Angiotensin-Converting Enzyme Inhibitor; ARBs: Angiotensin Receptor Blockers; ARNI: Angiotensin Receptor-Neprilysin Inhibitor BBs: Beta-Blockers; CCB: Calcium Channel Blocker; DAPT: dual antiplatelet therapy; DOAC: Direct Oral AntiCoagulant; PPI: Proton Pump Inhibitor, SGLT2i: Sodium–Glucose Cotransporter-2 Inhibitor; TZDs: Thiazolidinediones; VKA: Vitamin K Antagonist.

## Data Availability

The raw data supporting the conclusions of this article will be made available by the authors upon request, in accordance with the Ethical Committee’s data protection policy.

## References

[1] Feldman T., Kar S., Rinaldi M., Fail P., Hermiller J., Smalling R., Whitlow P.L., Gray W., Low R., Herrmann H.C. (2009). Percutaneous Mitral Repair with the MitraClip System. Safety and Midterm Durability in the Initial EVEREST (Endovascular Valve Edge-to-Edge REpair Study) Cohort. J. Am. Coll. Cardiol..

[2] Glower D.D., Kar S., Trento A., Lim D.S., Bajwa T., Quesada R., Whitlow P.L., Rinaldi M.J., Grayburn P., Mack M.J. (2014). Percutaneous mitral valve repair for mitral regurgitation in high-risk patients: Results of the EVEREST II study. J. Am. Coll. Cardiol..

[3] Nickenig G., Estevez-Loureiro R., Franzen O., Tamburino C., Vanderheyden M., Lüscher T.F., Moat N., Price S., Dall’ara G., Winter R. (2014). Percutaneous mitral valve edge-to-edge Repair: In-hospital results and 1-year follow-up of 628 patients of the 2011-2012 pilot European Sentinel Registry. J. Am. Coll. Cardiol..

[4] Feldman T., Foster E., Glower D.D., Kar S., Rinaldi M.J., Fail P.S., Smalling R.W., Siegel R., Rose G.A., Engeron E. (2011). Percutaneous repair or surgery for mitral regurgitation. N. Engl. J. Med..

[5] Puls M., Lubos E., Boekstegers P., von Bardeleben R.S., Ouarrak T., Butter C., Zuern C.S., Bekeredjian R., Sievert H., Nickenig G. (2016). One-year outcomes and predictors of mortality after MitraClip therapy in contemporary clinical practice: Results from the German transcatheter mitral valve interventions registry. Eur. Heart J..

[6] Stone G.W., Lindenfeld J., Abraham W.T., Kar S., Lim D.S., Mishell J.M., Whisenant B., Grayburn P.A., Rinaldi M., Kapadia S.R. (2018). Transcatheter Mitral-Valve Repair in Patients with Heart Failure. N. Engl. J. Med..

[7] Kar S., von Bardeleben R.S., Rottbauer W., Mahoney P., Price M.J., Grasso C., Williams M., Lurz P., Ahmed M., Hausleiter J. (2023). Contemporary Outcomes Following Transcatheter Edge-to-Edge Repair: 1-Year Results from the EXPAND Study. JACC Cardiovasc. Interv..

[8] von Bardeleben R.S., Mahoney P., Morse M.A., Price M.J., Denti P., Maisano F., Rogers J.H., Rinaldi M., De Marco F., Rollefson W. (2023). 1-Year Outcomes with Fourth-Generation Mitral Valve Transcatheter Edge-to-Edge Repair from the EXPAND G4 Study. JACC Cardiovasc. Interv..

[9] Stone G.W., Adams D.H., Abraham W.T., Kappetein A.P., Généreux P., Vranckx P., Mehran R., Kuck K.-H., Leon M.B., Piazza N. (2015). Clinical Trial Design Principles and Endpoint Definitions for Transcatheter Mitral Valve Repair and Replacement: Part 2: Endpoint Definitions A Consensus Document from the Mitral Valve Academic Research Consortium. J. Am. Coll. Cardiol..

[10] World Medical Association (2013). World Medical Association declaration of Helsinki: Ethical principles for medical research involving human subjects. JAMA.

[11] Vahanian A., Beyersdorf F., Praz F., Milojevic M., Baldus S., Bauersachs J., Capodanno D., Conradi L., De Bonis M., De Paulis R. (2022). 2021 ESC/EACTS Guidelines for the management of valvular heart disease. Eur. Heart J..

[12] Carrabba N., Migliorini A., Fumagalli C., Berteotti M., Matteo V., Cerillo A., Stefano P., Marchionni N., Valenti R. (2022). MitraClip implantation in real-world: Clinical relevance of different patterns of left ventricular remodeling. Hell. J. Cardiol..

[13] Kar S., Lim D.S., Fail P., Whisenant B., Rinaldi M.J., Grayburn P., Smalling R.W., Foster E., Weissman N.J., Feldman T. (2015). The everest II realism continued access study-outcomes at 1 year by mitral regurgitation etiology in non-high surgical risk patients. Catheter. Cardiovasc. Interv..

[14] Obadia J.-F., Messika-Zeitoun D., Leurent G., Iung B., Bonnet G., Piriou N., Lefèvre T., Piot C., Rouleau F., Carrié D. (2018). Percutaneous Repair or Medical Treatment for Secondary Mitral Regurgitation. N. Engl. J. Med..

[15] Rogers J.H., Asch F., Sorajja P., Mahoney P., Price M.J., Maisano F., Denti P., Morse M.A., Rinaldi M., Bedogni F. (2023). Expanding the Spectrum of TEER Suitability: Evidence from the EXPAND G4 Post Approval Study. JACC Cardiovasc. Interv..

[16] Ponikowski P., Friede T., von Bardeleben R.S., Butler J., Khan M.S., Diek M., Heinrich J., Geyer M., Placzek M., Ferrari R. (2024). Hospitalization of Symptomatic Patients with Heart Failure and Moderate to Severe Functional Mitral Regurgitation Treated with MitraClip: Insights from RESHAPE-HF2. J. Am. Coll. Cardiol..

[17] Falasconi G., Melillo F., Pannone L., Adamo M., Ronco F., Latib A., Rahgozar K., Carrabba N., Valenti R., Citro R. (2021). Use of edge-to-edge percutaneous mitral valve repair for severe mitral regurgitation in cardiogenic shock: A multicenter observational experience (MITRA-SHOCK study). Catheter. Cardiovasc. Interv..

[18] Stone G.W., Abraham W.T., Lindenfeld J., Kar S., Grayburn P.A., Lim D.S., Mishell J.M., Whisenant B., Rinaldi M., Kapadia S.R. (2023). Five-Year Follow-up after Transcatheter Repair of Secondary Mitral Regurgitation. N. Engl. J. Med..

[19] Mcmurray J.J.V., Packer M., Desai A.S., Gong J., Lefkowitz M.P., Rizkala A.R., Rouleau J.L., Shi V.C., Solomon S.D., Swedberg K. (2014). Angiotensin-neprilysin inhibition versus enalapril in heart failure. N. Engl. J. Med..

[20] Kang D.-H., Park S.-J., Shin S.-H., Hong G.-R., Lee S., Kim M.-S., Yun S.-C., Song J.-M., Park S.-W., Kim J.-J. (2019). Angiotensin Receptor Neprilysin Inhibitor for Functional Mitral Regurgitation: PRIME Study. Circulation.

[21] Januzzi J.L., Omar A.M.S., Liu Y., Murphy S., Butler J., Felker G.M., Piña I.L., Ward J., Solomon S., Contreras J. (2022). Association Between Sacubitril/Valsartan Initiation and Mitral Regurgitation Severity in Heart Failure with Reduced Ejection Fraction: The PROVE-HF Study. Circulation.

[22] McDonagh T.A., Metra M., Adamo M., Gardner R.S., Baumbach A., Böhm M., Burri H., Butler J., Čelutkienė J., Chioncel O. (2021). 2021 ESC Guidelines for the diagnosis and treatment of acute and chronic heart failure. Eur. Heart J..

